# A case report of primary unilateral adrenal NK/T cell lymphoma: good clinical outcome with trimodality treatment

**DOI:** 10.1186/s12885-016-3019-1

**Published:** 2017-01-05

**Authors:** Liu Hu, Weimin Xu, Mingwei Wang, Pan Wang, Guang Han, Chi Lin

**Affiliations:** 1 Department of Radiation Oncology, Hubei Cancer Hospital, Tongji Medical College, Huazhong University of Science and Technology, Wuhan, 430079 China; 2 Department of ENT, Wuhan Puai Hospital, Tongji Medical College, Huazhong University of Science and Technology, Wuhan, 430034 China; 3 Department of Pathology, Hubei Cancer Hospital, Tongji Medical College, Huazhong University of Science and Technology, Wuhan, 430079 China; 4 Department of Dermatology, Wuhan Medical & Healthcare Center for Women and Children, Wuhan, 430015 China; 5 Department of Oncology, Renmin Hospital of Wuhan University, Wuhan, 430060 China; 6 Department of Radiation Oncology, University of Nebraska Medical Center, Omaha, NE 68198 USA

**Keywords:** Primary natural killer/T cell lymphoma, Adrenal glands, Trimodality treatment

## Abstract

**Background:**

Primary natural killer (NK)/T cell lymphoma of adrenal glands is an extremely rare tumor with aggressive clinical behavior. There have only been a few cases reported worldwide and the highest reported survival was 90 days. We report the first case of primary unilateral adrenal NK/T cell lymphoma in China with good outcome.

**Case presentation:**

This is a 28-year-old man who presented with abdominal pain and was found to have a large mass on the left adrenal and the top of the renal region. The patient underwent surgical resection and the pathology revealed primary adrenal NK/T cell lymphoma. He received adjuvant sandwich therapy encompassing sequential chemotherapy, radiotherapy and chemotherapy. The patient remains clinically and symptomatically disease-free with over two years follow up.

**Conclusion:**

Given the rarity of this disease, there is limited experience with regard to its diagnosis and treatment. This case report will add to the scant literature on this tumor and will be useful for the differential diagnosis and treatment of adrenal disease.

## Background

There have been seven reported cases of NK/T cell lymphoma involving adrenal masses worldwide [1–7]. Among them, only one was primary adrenal NK/T cell lymphoma [2]. There have been no reported cases of the primary adrenal NK/T-cell lymphoma in China. Anatomically, it usually originates in the nasal cavity/nasopharynx and invades the surrounding tissues. Sometimes, it can arise in other organs including the skin, spleen/liver, or the gastrointestinal tract [8]. Here, we report a primary unilateral adrenal NK/T cell lymphoma with subsequent management. Although the longest survival was reported to be 90 days for adrenal NK/T cell lymphoma, our patient has been disease-free for 26 months.

## Case presentation

### Case report

**Fig. 1:**
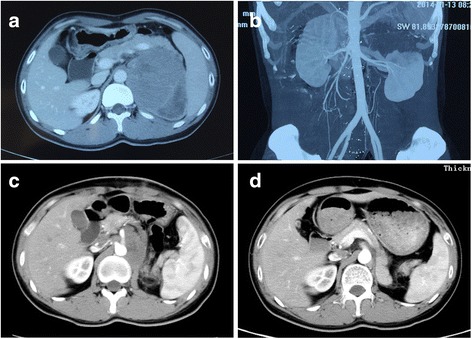
Abdominal CT scans. An abdominal axial CT scan indicates a large left adrenal mass that had invaded retroperitoneal cavity before surgery (**a**). A reconstructed coronal CT image shows that the top of left kidney was invaded by the tumor (**b**). An abdominal axial CT scan shows a residual mass after the surgery (**c**). An abdominal axial CT scan shows a complete response after the postoperative chemotherapy with four cycles of CHOP (**d**)

**Fig. 2:**
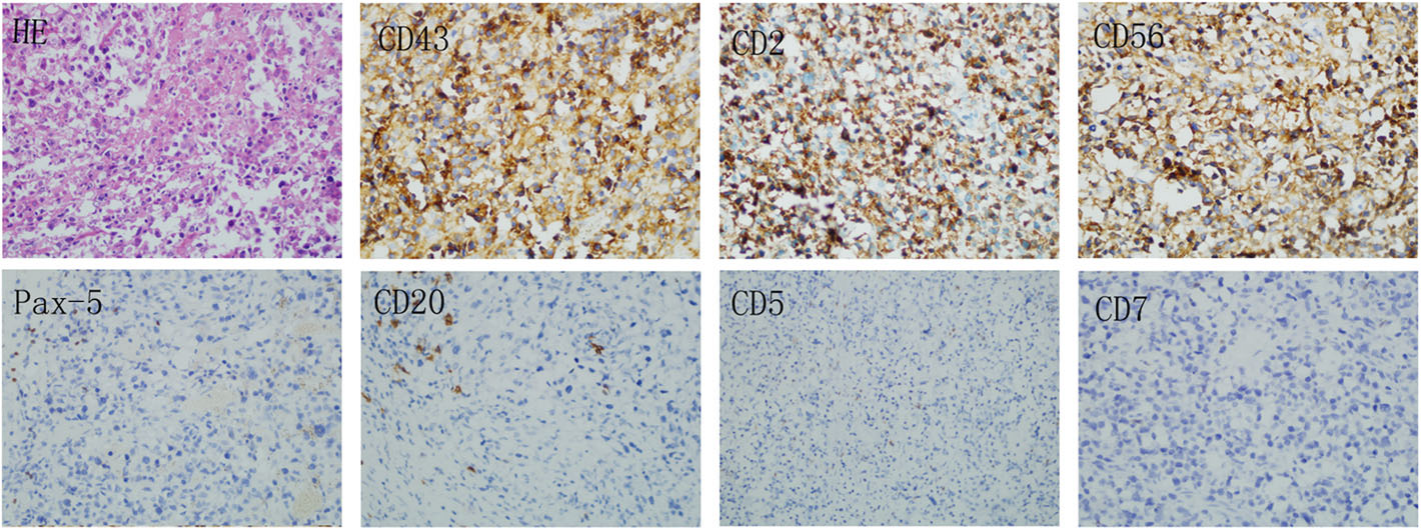
Histological and immunohistochemical analysis. Neoplastic infiltrate of relatively pleomorphic lymphoid cells with scanty cytoplasm, irregular nuclear contour, and prominent nucleoli (hematoxylin and eosin, magnification × 400). Immunohistochemical staining demonstrates positive (magnification × 400) for CD43, CD2, CD56, but negative (magnification × 200) for CD20, CD5, CD7, Pax-5

**Fig. 3:**
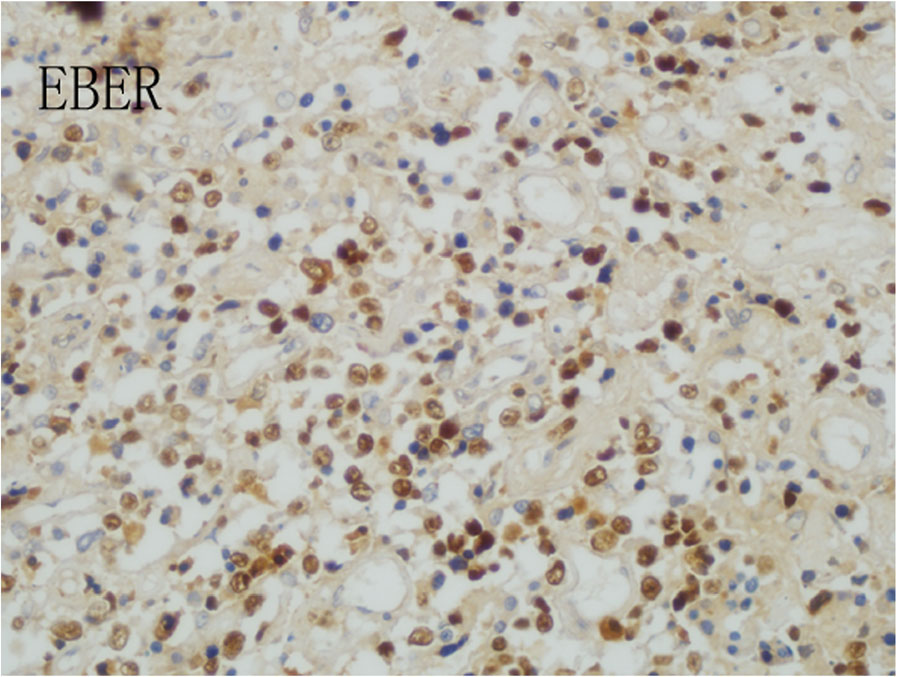
The expression of EBER. Chromogenic in situ hybridization (CISH) was positive for the EBER gene. (magnification × 400)

A 28-year-old male experienced abdominal pain and a mass on the left side of the abdomen. Color duplex ultrasonogram and subsequent computed tomography (CT) scan revealed a 10 × 9 cm adrenal mass invading the top of left renal and partial of retroperitoneal region (Fig. 1. a and b). He underwent surgical removal of the mass in an outside hospital. The pathology specimens were evaluated in our hospital which showed neoplastic infiltration of pleomorphic lymphoid cells with scanty cytoplasm, irregular nuclear contour, and prominent nucleoli. Immunohistochemistry analysis showed that the tumor cells were positive for CD2, Bcl-2, CD43, CD56, negative for CD20, CD4, CD5, CD7, CD8, CD138, PCK, HMB45, Des, Pax-5, Bcl-6, Vim, CD34, TdT, MPO and equivocal for CD3. Ki67 labelling index was 40% (Fig. 2). Chromogenic in situ hybridization (CISH) was positive for the EBER gene (Fig. 3). These are consistent with NK/T-cell lymphoma.

After the surgery, the patient was without any systemic symptoms. He did have a 10 kg weight loss secondary to the removal of the majority of the large mass and the poor postoperative nutrition support. The postoperative physical examination was unremarkable. A postoperative CT scan of the abdomen showed a residual tumor, measuring 2.7 × 5.3 cm at the left side of retroperitoneum with the absence of left adrenal gland and kidney (Fig. 1. c). The bone marrow biopsy was negative. Nasal magnetic resonance imaging (MRI) showed normal findings. The contrast CT scans of the neck, chest and pelvis had been done and showed normal tissue structure. Primary unilateral adrenal NK/T cell lymphoma was diagnosed. He received four cycles of chemotherapy with cyclophophamide, doxorubicin, vincristine and prednisolone (CHOP). A post-chemotherapy CT scan revealed no residual disease (Fig. 1. d). He then underwent a course of consolidated intensity-modulated radiotherapy to the surgical bed to a dose of 50Gy in 25 fractions. Post-radiotherapy, the patient received two more cycles of CHOP. The patient tolerated treatment well without significant side effects. At the 26 months follow up point, the patient remains clinically and symptomatically disease-free.

## Discussion

Primary adrenal lymphoma or lymphoma involving mainly the adrenal gland without the regional lymph node involvement is a very rare extra-nodal lymphoma. Patients with adrenal lymphoma may present with systemic symptoms including fever, fatigueand weight loss [9–11]. Primary adrenal lymphoma can be discovered incidentally on abdominal imaging. Differential diagnoses include cortical adenoma, pheochromocytoma, metastatic-disease, and adrenocorticotropic hormone (ACTH) dependent Cushing’s syndrome [12, 13]. The definitive diagnosis of primary adrenal lymphoma is made by an image-guided percutaneous biopsy, surgical exploration, or a postmortem examination [1, 2, 14]. Histopathologically, the most common type of primary adrenal lymphoma is B-cell type, mainly diffuse large B-cell lymphoma. Although involvement of the adrenal glands can be seen in disseminated disease, primary NK/T cell lymphoma in the adrenal gland as an initial presentation is extremely rare. The NK/T cell lymphomas are aggressive lymphoproliferative disorders derived from either activated NK cells or cytotoxic T cells. The 2008 World Organization classification system categorized NK–cell malignancies into three types: extra-nodal NK/T-cell lymphoma (nasal and non-nasal), aggressive NK-cell leukemia and chronic lymphoproliferative disorder of NK cells [15]. NK/T-cell lymphomas demonstrate vascular destruction with prominent necrosis. Immunophenotypically, they express CD2 and CD56. NK/T-cell lymphomas are strongly associated with Epstein-Barr virus (EBV), suggesting a pathogenic role of this virus [16, 17]. In this case, the patient’s tumor was positive for CD2, CD56 and EBER.

NK/T cell lymphoma is an extra-nodal lymphoma with predilection for the nasal cavity, nasopharynx, skin, and the other extra-nodal sites. The primary adrenal NK/Tcell lymphoma is exceedingly uncommon. In the review of the literature, we found only seven reported cases of NK/T cell lymphoma presenting as adrenal masses. Among these cases, the adrenal mass was the only involved site in one case [9]. The rest involved multiple organs, including central nervous system (CNS), paranasal sinus, kidney, bone marrow, etc. [1, 3–7]. Tissue diagnosis was made from the adrenal mass in four cases through adrenalectomy, CT-guided biopsy and autopsy specimen [1–3, 16], from cervical lymph nodes or nasal mass in two cases. There was no pathologic diagnosis in one case (the diagnosis was made per imaging study). The ages of the patients ranged from 17 to 76. Among these cases, the highly aggressive nature of the lymphoma and its rapid clinical progression were reported and the long-term prognosis has not been observed (Table. 1).

**Table 1:** Summary of patients with adrenal NK/T cell lymphoma

Authors	Year	Age	Sex	Organs invasion	Bilateral or unilateral	Type of specimen	Treatment	Survival	EBV positive
Dunning et al. [1]	2009	65	male	Bone marrow	unilateral	CT-guided biopsy	High-dose intravenous dexamethasone	(Died) few days after the final pathologic diagnosis	Yes
Thompson et al. [2]	2007	35	male	None	bilateral	Adrenalectomy	Surgery and chemotherapy (CHOP)	(Died) 90 days after the final pathologic diagnosis	Yes
Mizoguchi et al. [3]	2005	17	male	No mention	bilateral	Autopsy specimen	Immunosuppression therapy or plasma exchange	(Died) 4 days after admission	Yes
Toba et al. [4]	2008	76	female	Nasal, pleura	bilateral	Biopsy of the nasal tumor	Chemotherapy (THP-COP)	(Died) 60 days after admission	No
Nagireddy et al. [5]	2011	70	male	Meninges, left middle cranial fossa, paranasal sinus, breast, lungs, pleura, kidney, and prostate	bilateral	CT-guided biopsy	Chemotherapy (CHOP)	(Died) one month later while on hospice	Yes
Kang et al. [6]	2011	29	male	Multiple cervical, mediastinal and abdominal lymph nodes	bilateral	Excisional biopsy of cervical lymph nodes	Chemotherapy (ifosfamide, methotrexate, etoposide and prednisolone)	(Died) 59 days after his first admission	No mention
Jin et al. [7]	2005	37	male	Oral cavity, pharyngel, spleen, lymph nodes	bilateral	None	Chemotherapy (CHOP)	(Died) 21 days after admission	No mention
Our case	2014	28	male	Kidney	unilateral	Adrenalectomy	Surgery, chemotherapy (CHOP) and radiotherapy	(Alive) 26 months after treatment	Yes

Because of the rarity of this disease, the standard therapies have not been defined. The treatments have included various combinations of surgery, chemotherapy, and radiation. In the reported cases, four patients received chemotherapy, one patient received surgery and chemotherapy; one patient received high dose intravenous dexamethasone, and one patient received immunosuppression therapy. CHOP was the most common used chemotherapy regimen in these reported cases. The longest reported survival was 90 days in a patient who received surgery and chemotherapy. Our patient underwent surgery, postoperative chemotherapy with 4 cycles of CHOP followed by radiotherapy. Two more cycles of CHOP were administered afterwards. It should be noted that the surgery was performed prior to the patient’s visit at our hospital. If a biopsy sufficed, then surgery would not be necessary, given that these tumors are generally sensitive to chemotherapy and radiation therapy. Because of the residual tumor at the left side of the retroperitoneum, postoperative radiation therapy was recommended. In terms of chemotherapy regimen, SMILE (dexamethasone, methotrexate, ifosfamide, L-asparaginase and etoposide) is a standard chemotherapy regimen for NK/T lymphoma patients in the western countries. However, there was a shortage of L-asparaginase in our province in the end of 2013, when this patient was first treated. Therefore, this patient was treated with CHOP according to the previous case reports [2, 5, 7]. As a side note, SMILE has been used for NK/T lymphoma patients in our province since mid-2014.

Our patient remains disease-free with a follow up of over two years. We list three possible reasons for the longer disease-free survival for our patient: (1) the NK/T cell lymphoma only involved one adrenal gland; (2) the NK/T cell lymphoma did not involve other organs, except for the adjacent kidney; and (3) the utilization of trimodality therapy.

## Conclusion

In conclusion, primary adrenal NK/T cell lymphoma is an exceedingly uncommon neoplasm and the outcomes for it are generally poor. We present the first case of a primary adrenal NK/T cell lymphoma in China with over 2-year disease-free survival treated with trimodality therapy. NK/T cell lymphoma should be considered in the differential diagnosis of a rapidly enlarging adrenal mass. As reports of such cases increase and our understanding of the disease improves, we anticipate more effective treatment for these patients.

## Abbreviations

ACTH: Adrenocorticotropic hormone
CHOP: Cyclophosphamide, doxorubicin, vincristine and prednisolone
CISH: Chromogenic in situ hybridization
CNS: Central nervous system
CT: Computed tomography
EBV: Epstein-Barr virus
MRI: Magnetic resonance imaging
NK/T cell: Natural killer/T cell
SMILE: dexamethasone, methotrexate, ifosfamide, L-asparaginase and etoposide
